# Predictors of in-hospital appendiceal perforation in patients with non- perforated acute appendicitis with appendicolithiasis at presentation

**DOI:** 10.1186/s12893-023-02210-4

**Published:** 2023-10-18

**Authors:** Amir H. Sohail, Hazim Hakmi, Koral Cohen, Joshua C. Hurwitz, Jasmine Brite, Sawyer Cimaroli, Harry Tsou, Michael Khalife, James Maurer, Matthew Symer

**Affiliations:** 1 Department of Surgery, NYU Langone Long Island, 222 Station Plaza N. Suite 300, Mineola, NY 11501 USA; 2 NYU Long Island School of Medicine, Mineola, NY 11501 USA; 3 Mount Sinai South Nassau Hospital Department of Surgery, Oceanside, NY 11572 USA

**Keywords:** Acute Appendicitis, Perforated Appedicitis, Appendectomy, Risk Factors, Appendicolith

## Abstract

**Introduction:**

Appendicolithiasis is a risk factor for perforated acute appendicitis. There is limited inpatient data on predictors of progression in appendicolithiasis-associated non-perforated acute appendicitis.

**Methods:**

We identified adults presenting with appendicolithiasis-associated non-perforated acute appendicitis (on computed tomography) who underwent appendectomy. Logistic regression was used to investigate predictors of in-hospital perforation (on histopathology).

**Results:**

296 patients with appendicolithiasis-associated non-perforated acute appendicitis were identified; 48 (16.2%) had perforation on histopathology. Mean (standard deviation [SD]) age was 39 (14.9) years. The mean (SD) length of stay (LOS) was 1.5 (1.8) days. LOS was significantly longer with perforated (mean [SD]: 3.0 [3.1] days) vs. non-perforated (mean [SD]: 1.2 [1.2] days) appendicitis (*p* < 0.001). On multivariate analysis, in-hospital perforation was associated with age > 65 years (OR 5.4, 95% CI: 1.4- 22.2; *p* = 0.015), BMI > 30 kg/m2 (OR 3.5, 95% CI: 1.3–8.9; *p* = 0.011), hyponatremia (OR 3.6, 95% CI: 1.3–9.8; *p* = 0.012). There was no significant association with age 25–65 years, gender, race, steroids, time-to- surgery, neutrophil percentage, or leukocyte count.

**Conclusion:**

Geriatric age, obesity, and hyponatremia are associated with progression to perforation in appendicolithiasis-associated non-perforated acute appendicitis.

## Introduction

Acute appendicitis is one of the most common surgical pathologies. An estimated 280,000 appendectomies are performed in the United States annually [1]. Since Claudius Amyand performed the first appendectomy in 1735, methods for diagnosis, surgical technique, and perioperative management of acute appendicitis have drastically been refined. With the advent and adoption of advanced diagnostic imaging modalities such as computed tomography, identification of pathologic features such as appendicolithiasis, and complications such as phlegmon, abscess, and perforation is possible prior to histopathologic analysis of surgical specimens.

Prompt identification of patients with uncomplicated appendicitis at risk for perforation is crucial, and may prevent progression to complicated appendicitis and the associated increase in surgical morbidity and mortality. Evidence suggests that factors associated with perforated appendicitis include male gender, advanced age, appendicolithiasis and presence of medical comorbidities [2, 3]. Interestingly, within the appendicolithiasis associated non-perforated appendicitis subgroup, there is a paucity of data regarding factors associated with the development of appendiceal perforation.

We aimed to investigate factors associated with the development of perforated appendicitis in patients who presented to our institution with non-perforated appendicitis and appendicolith on initial computed tomography.

## Methods

We retrospectively reviewed electronic medical records for patients who presented to our institution (New York University Langone Health) with acute appendicitis and subsequently underwent appendectomy on the same admission over a period of 7 years. Institutional Review Board approval was obtained for this study from NYU Langone Long Island School of Medicine. Inclusion criteria were age ≥ 18 years, diagnosis of acute, non-perforated appendicitis with appendicolith on admission computed tomography, and subsequent appendectomy during index admission. All patients with perforated appendicitis on initial imaging at presentation were excluded from analyses.

Information on demographics (age, sex, race), medications (steroid use), anthropometric measures (height and weight, to obtain Body Mass Index [BMI]), laboratory investigations on admission (serum sodium, white blood cell count, polymorphonuclear neutrophils [PMN]), hospital course (interval between presentation and appendectomy, length of hospital stay, intensive care requirements), and histopathology findings (presence of perforation) was retrieved. Hyponatremia was defined as serum sodium of < 135 mmol/L.

All continuous variables were demonstrated as mean and standard deviation (SD), unless otherwise noted, and percentage was obtained for all binary variables. We conducted univariate logistic regression to investigate association of each variable with perforation on histopathology. Multivariate logistic regression was used to investigate factors associated with in-hospital appendiceal perforation. Sensitivity analyses were performed by excluding patients with hyponatremia at presentation (since previous data shows that perforated appendicitis patients are often hyponatremic) and conducting multivariate logistic regression.

Patients with missing data were excluded from analyses. All analyses were conducted using R: A language and environment for statistical computing (R Core Team (2019). Vienna, Austria:R Foundation for Statistical Computing, Vienna, Austria) [4–6]. A *p*-value < 0.05 was defined as significant.

## Results

During the study period, we identified 296 admissions and procedures for appendicolithiasis associated with non-perforated acute appendicitis with complete data on all key variables. Out of the 296 patients, 48 patients (16.2%) were found to have perforated appendicitis on histopathology. Participant demographic and baseline data are displayed in Table 1. This was a predominantly young patient cohort with limited health comorbidities. The mean (SD) age for patients was 39 (14.9) years. The majority of patients were male (*n* = 172; 58.1%) and white race (*n* = 198; 66.9%). The patient population had a low prevalence of hypertension (*n* = 17, 5.7%) and diabetes mellitus (*n* = 9, 3.0%). Mean (SD) BMI for study participants was 24.7 (4.4) kg/m2. Two percent of (*n* = 6) participants were on steroids at the time of presentation.

**Table 1:**
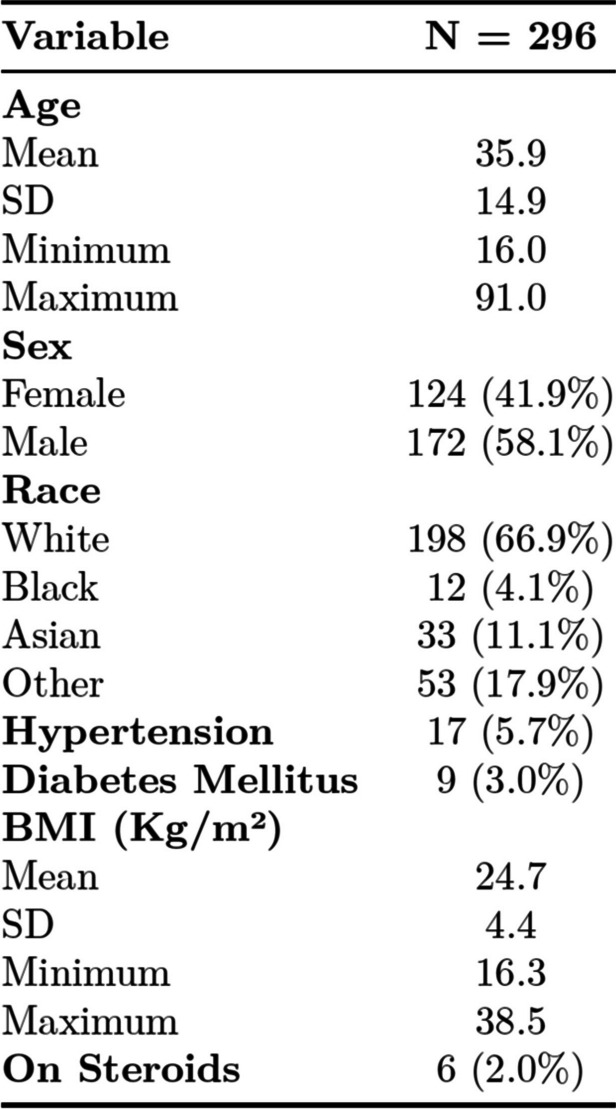
Patient demographics

Table 2 details preoperative and postoperative findings for the study population. Of note, one- third (*n* = 9) of hyponatremic patients at presentation (*n* = 27) were found to have perforated appendicitis on histopathology. The mean (SD) time to the operating room was 12.1 (6.2) hours. The mean (SD) length of hospital stay for study participants was 1.5 (1.8) days. Mean (SD) length of hospital stay was significantly longer in the perforated acute appendicitis (3.0, 3.1 days) group as compared to non- perforated (1.2, 1.2 days) acute appendicitis participants (*p* < 0.001).

**Table 2:**
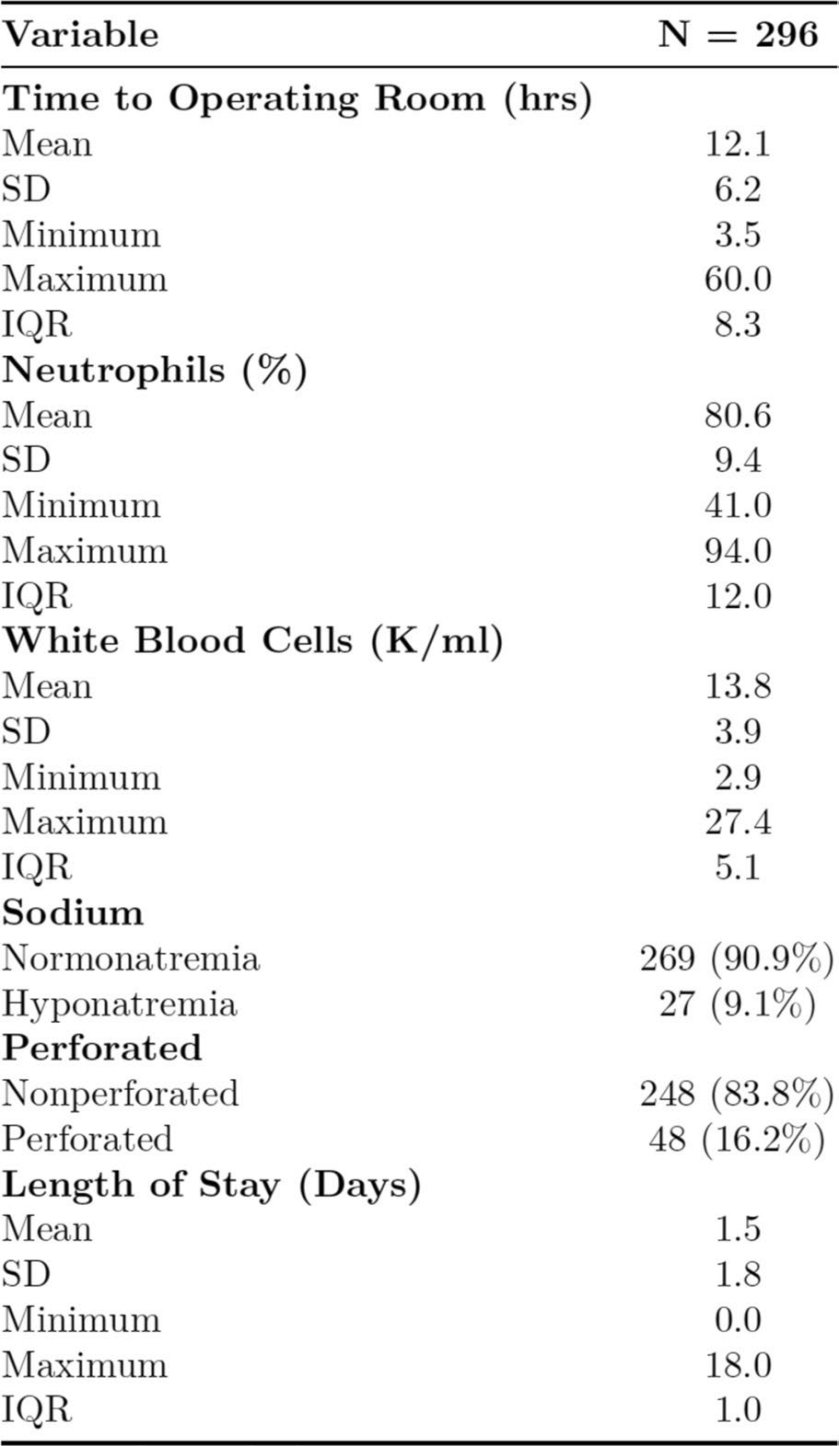
Peroperative and operative findings in patients with non-perforated acute appendicitis with appendicolith in computed tomography

On univariate analyses, factors associated with perforated acute appendicitis included age > 65 years versus 18–25 years (OR 4.12; 95% CI: 1.36—12.48), hypertension (OR: 3.08; 95% CI: 1.01—8.56),BMI > 30 kg/m2 (OR: 2.78; 95% CI: 1.22—6.04), and hyponatremia at presentation (OR: 2.95; 95% CI: 1.19—6.90). Age 25–65 years (versus 18–25 years), sex, race, diabetes mellitus, steroid use, time from presentation to operating room, neutrophil percentage, leukocyte count were not significantly associated with in-hospital appendiceal perforation (*p*-value > 0.05 for all variables).

On multivariate analysis, in-hospital perforation was significantly associated with age > 65 years (OR 5.44, 95% CI: 1.38–22.1; *p* = 0.015), BMI > 30 (OR 3.50, 95% CI: 1.31–8.85; *p* = 0.011), and hyponatremia (OR 3.63, 95% CI: 1.29–9.83; *p* = 0.012). See Table 3 for details of multivariate analysis. Perforation was not significantly associated with age 25–65 years old, gender, race, steroid use, time to operating room, neutrophil percentage or WBC count (*p* > 0.05 for all covariates).

**Table 3:**
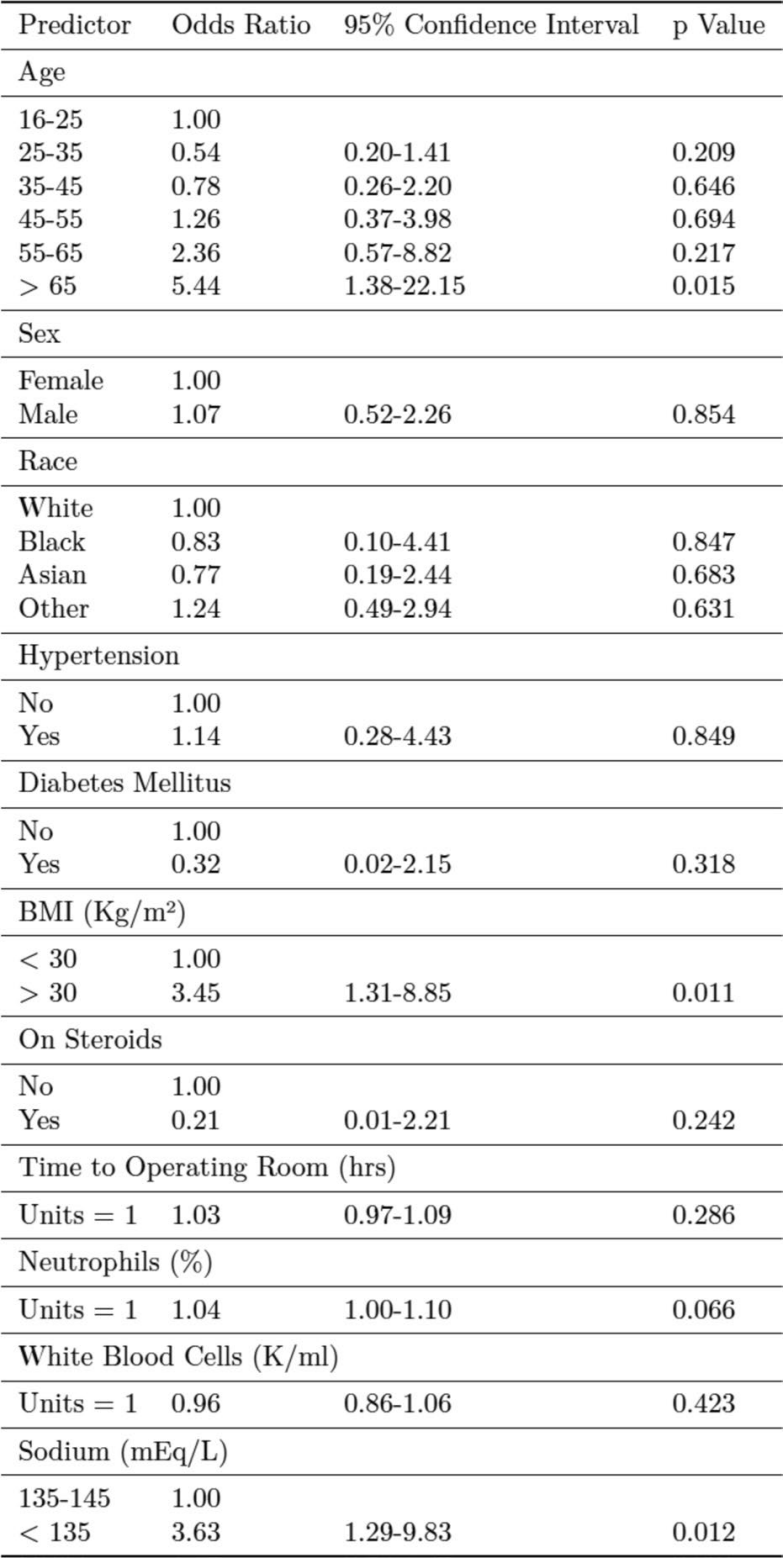
Factors affecting likelihood of perforation

Sensitivity analyses, after exclusion of patients with hyponatremia at presentation (*n* = 27), did not significantly alter results. Advanced age (OR 9.03; 95% CI 1.95—44.70) and BMI > 30 kg/m2 (OR 3.46; 95% CI 1.25—9.29) were both associated with increased risk of perforation on multivariate analysis.

## Discussion

Our results show that 16.2% of participants had in-hospital progression to perforation, an estimate which has never been reported previously. Further, our study shows that in patients with appendicolithiasis and non-perforated appendicitis on initial imaging, patient factors, such as geriatric age and obesity are significantly associated with in-hospital appendiceal perforation. Laboratory finding of hyponatremia is also predictive of in-hospital perforation.

Appendicolithiasis has been reported to be found in 38.7% of patients with acute appendicitis [7]. Moreover, appendicolithiasis is associated with a higher rate of appendiceal rupture/abscess formation and a higher failure rate of conservative management with antibiotics [8]. Malinen et al. investigated the histopathological features of acute appendicitis with and without appendicolithiasis and found signs of severe acute inflammation in ~ 50% of patients with an appendicolith vs only ~ 15% of those without appendicolith. Micro-abscesses formation and epithelial damage were twofold in the appendicolith group [8–10]. However, factors contributing to this progression in this sub-population are poorly understood. To the best of our knowledge, this is the first study investigating factors associated with in-hospital progression to perforation in patients with non- perforated appendicolithiasis-associated acute appendicitis at presentation. It is noteworthy that our results are largely consistent with previous data on factors associated with appendiceal perforation. For instance, Walker et al. showed that in general geriatric patients had a higher risk of perforation (OR 1.04; 95% CI 1.02–1.05).

While time to appendectomy has previously been shown to be associated with appendiceal perforation, our study did not find a significant association. This is possibly a result of the fact that most patients in our study underwent an appendectomy within 24 h of presentation. Further, it is also possible that our study was underpowered to detect this association.

It has been suggested in literature that hyponatremia at presentation is indicative of perforation rather than being an early predictor of impending perforation. Thus, it can be argued that the 27 patients with hyponatremia at presentation in our study population already had perforated appendicitis at the time of presentation and the perforation was missed on computed tomography due to limited sensitivity. However, histopathological examination showed perforation in only one-third (*n* = 9) of these patients. Further, our sensitivity analysis after excluding hyponatremic patients did not change our final results; geriatric age and obesity were still significantly associated with in-hospital progression to perforation.

Diagnostic imaging modalities for diagnosis of acute appendicitis include ultrasonography, MRI or CT. CT imaging has a sensitivity of ~ 90% in comparison to MRI which has a sensitivity of ~ 97% in diagnosing acute appendicitis [11]. However, the radiologic diagnosis of complicated appendicitis is more challenging. A meta-analysis of CT features for differentiating complicated and uncomplicated appendicitis found ten CT features for differentiating complicated appendicitis, nine of which showed high specificity, and one of which was highly sensitive [12].

The major limitations of our study include its retrospective, observational design with its inherent biases. The sensitivity of computed tomography in detecting perforation (at the time of initial presentation) is variable, and especially dependent upon the interpreter; thus some of the participants with perforation on histopathology may have already had perforated appendicitis at the time of initial imaging. Further, due to the limited number of patients with perforation on histopathology, our study might be underpowered to detect certain associations, such as that with time to operating room or steroid use. Due to absence of information, we were unable to assess the utility of other biochemical markers, such as C-reactive protein (CRP), hyperbilirubinemia and hyperfibrinogenemia, in predicting appendiceal perforation in our population [13–16].

## Conclusion

A sizable proportion of patients presenting with appendicolithiasis-associated non-perforated acute appendicitis on initial imaging experience in-hospital appendiceal perforation (16.2% in our data). Perforation increases risk of postoperative adverse outcomes including increased hospital length of stay. Factors associated with in-hospital perforation include geriatric age, obesity, and hyponatremia.

## Data Availability

The datasets generated and analysed during the current study are not publicly available due institutional policy, but are available from the corresponding author on reasonable request.
